# Biocatalysis of Platycoside E and Platycodin D3 Using Fungal Extracellular β-Glucosidase Responsible for Rapid Platycodin D Production

**DOI:** 10.3390/ijms19092671

**Published:** 2018-09-08

**Authors:** Hyung Jin Ahn, Hyun Ju You, Myung Su Park, Tony V. Johnston, Seockmo Ku, Geun Eog Ji

**Affiliations:** 1 Department of Food and Nutrition, Research Institute of Human Ecology, Seoul National University, Seoul 08826, Korea; ahnjin2000@snu.ac.kr; 2 Center for Human and Environmental Microbiome, Institute of Health and Environment, Seoul National University, Seoul 08826, Korea; dhlover1@snu.ac.kr; 3 Department of Hotel Culinary Arts, Yeonsung University, Anyang 14001, Korea; mspark@yeonsung.ac.kr; 4 Fermentation Science Program, School of Agriculture, College of Basic and Applied Sciences, Middle Tennessee State University, Murfreesboro, TN 37132, USA; tony.johnston@mtsu.edu; 5 Research Center, BIFIDO Co., Ltd., Hongcheon 25117, Korea

**Keywords:** platycodi radix, *platycodon grandiflorum*, biotransformation, biocatalysis, *aspergillus usamii*, β-glucosidase, phytochemicals, platycoside E, platycodin D3, platycodin D

## Abstract

Platycodi radix (i.e., *Platycodon grandiflorum* root) products (e.g., tea, cosmetics, and herbal supplements) are popular in East Asian nutraceutical markets due to their reported health benefits and positive consumer perceptions. Platycosides are the key drivers of Platycodi radixes’ biofunctional effects; their nutraceutical and pharmaceutical activities are primarily related to the number and varieties of sugar side-chains. Among the various platycosides, platycodin D is a major saponin that demonstrates various nutraceutical activities. Therefore, the development of a novel technology to increase the total platycodin D content in Platycodi radix extract is important, not only for consumers’ health benefits but also producers’ commercial applications and manufacturing cost reduction. It has been reported that hydrolysis of platycoside sugar moieties significantly modifies the compound’s biofunctionality. Platycodi radix extract naturally contains two major platycodin D precursors (platycoside E and platycodin D3) which can be enzymatically converted to platycodin D via β-d-glucosidase hydrolysis. Despite evidence that platycodin D precursors can be changed to platycodin D in the Platycodi radix plant, there is little research on increasing platycodin D concentrations during processing. In this work, platycodin D levels in Platycodi radix extracts were significantly increased via extracellular *Aspergillus usamii* β-d-glucosidase (*n* = 3, *p* < 0.001). To increase the extracellular β-d-glucosidase activity, *A. usamii* was cultivated in a culture media containing cellobiose as its major carbon source. The optimal pH and temperature of the fungal β-d-glucosidase were 6.0 and 40.0 °C, respectively. Extracellular *A. usamii* β-d-glucosidase successfully converted more than 99.9% (*w/v*, *n* = 3, *p* < 0.001) of platycoside E and platycodin D3 into platycodin D within 2 h under optimal conditions. The maximum level of platycodin D was 0.4 mM. Following the biotransformation process, the platycodin D was recovered using preparatory High Performance Liquid Chromatography (HPLC) and applied to in vitro assays to evaluate its quality. Platycodin D separated from the Platycodi radix immediately following the bioconversion process showed significant anti-inflammatory effects from the Lipopolysaccharide (LPS)-induced macrophage inflammatory responses with decreased nitrite and IL-6 production (*n* = 3, *p* < 0.001). Taken together, these results provide evidence that biocatalysis of Platycodi radix extracts with *A. usamii* may be used as an efficient method of platycodin D-enriched extract production and novel Platycodi radix products may thereby be created.

## 1. Introduction

Platycodi radix (PR), the *Platycodon grandiflorum* root, has traditionally been used in popular herbal medicine for the prevention and treatment of cold-related symptoms (e.g., coughs, phlegm, tonsillitis, asthma and bronchitis) in various East Asian countries [1,2]. Modern analytical chemists and food scientists have verified that triterpenoid saponins known as platycosides are the major phytochemicals of PR’s bio-functionality as a plant medicine [3]. About 69 platycoside varieties have been identified as key functional substances of PR [4]. According to Ha et al. [5], platycosides account for only 2% of PR; however, the total platycoside content of PR may be dependent on *Platycodon grandiflorum*’s soil, weather, cultivation method, and years of growth [6]. PR’s nutraceutical and pharmaceutical properties vary widely according to the type, position, and number of sugars on the saponin molecules [7].

Among the various platycosides, platycodin D (PD, chemical formula: C_57_H_92_O_28_; formal name: 3β-(β-d-glucopyranosyloxy)-2β,16α,23,24-tetrahydroxy-*O*-d-apio-β-d-furanosyl-(1→3)-*O*-β-d-xylopyranosyl-(1→4)-*O*-6-deoxy-olean-12-en-28-oic acid; Chemical abstracts service number: 58479-68-8; Molecular weight: 1225.34), consisting of a main oleanane type backbone with two R groups (i.e., 3-Glc, and 28-*O*-Api-Xyl-Rha-Ara), is regarded as the key bio-active molecule of PR [8,9]. Previous research has reported that platycodin D reduced the risk of inflammation [10], obesity [11], Hepatitis C viral infection [12], allergy-related diseases [13], tumorigenicity [14] and the development of cancers (i.e., liver, ovarian, breast, gastric and leukemia) [15,16,17,18,19]. These platycodin D studies demonstrated that platycodin D-enriched products can be used not only to alleviate cold-related symptoms but also to prevent chronic and fatal diseases. The platycodin D level in commercial products is the key factor used to appraise product quality and meet consumers’ needs [1]. Multiple chemical precursors with the potential to transform into platycodin D are present in PR [3]. Theoretically, a technique that efficiently transforms the chemical structures of these precursors without degrading the existing platycodin D would increase the total platycodin D content of PR products. Such a technique would ultimately contribute to improving the quality of the product and its pharmacological effects. 

Enzymatic bio-conversion has been extensively utilized to structurally transform plant saponins (e.g., ginsenoside, wogonoside, daidzin and genistin) [20,21,22,23,24]. Unconjugated forms of glycosides (also known as aglycones and deglycosylated saponins) are most desirable, and the use of an edible microbial enzyme to execute the bioconversion provides for stereospecific and sustainable reactions and may offer eco-friendly and time-cost effective processing attributes [25]. Two major platycodin D precursors (i.e., platycoside E (PE) and platycodin D3 (PD3)) can be converted to PD using β-d-glucosidase (EC 3.2.1.21; β-d-glucopyranoside glucohydrolase; gentiobiase; cellobiase) hydrolysis [5,26,27]. Despite the existence of two major PR precursors in the source plant, there is little research on increasing PD production during processing. Accordingly, the principal objective of our research is the development of a novel protocol for rapid PD production using fungal biocatalysis of PD precursors during processing. After PD production, we conducted qualitative (liquid chromatography/mass spectrometry (LC/MS) and cell inflammatory assays) and quantitative (high-performance liquid chromatography (HPLC)) analysis to demonstrate the practicality of our protocol, which allowed us to consistently obtain high PD yields via PE and PD3 conversion. 

## 2. Results and Discussions

### 2.1. Microbial Screening

Among the various food-grade edible microorganisms, *Aspergillus*, *Lactobacillus,* and *Bifidobacterium* spp. are considered by the food industry as effective β-d-glucosidase producers [28]. Many research groups and individual researchers have tried to cultivate microorganisms in media using glucose as the major carbon source for active proliferation [23,29,30]. Glucose is one of the simplest nutrients used by microorganisms to support rapid microbial growth [23]. In the vast majority of documented cases, the most favored microbial sugar source is glucose. Glucose serves in industry as a useful metabolic precursor for obtaining large quantities of cell biomass [23]. However, if producers aim to produce enzymes rather than cell biomass, their microbial culture strategy must be changed. The addition of glucose to microbial media can significantly reduce the production of the desired microbial enzyme (i.e., β-d-glucosidase) due to catabolic repression [31]. In contrast, other research using in-house media containing cellobiose as a carbon source showed that some microorganisms have the potential to express substantial levels of β-d-glucosidase [22]. Therefore, in this study, six probiotics (*Lactobacillus* and *Bifidobacterium* spp.) and five *Aspergillus* strains were cultured in glucose-free culture media. Cellobiose was added as the major carbon source to enhance microbial β-d-glucosidase activity. 

Bacterial and fungal cell extracts and media samples were used to evaluate their β-d-glucosidase-producing abilities via *p*-nitrophenyl-β-d-glucopyranoside (pNP, artificial substrate) and platycoside catalysis. Crude microbial enzyme preparations exhibiting substantial catalytic activity demonstrate a light or dark yellow color (450 nm) during enzymatic hydrolysis due to the release of *p*-nitrophenol molecules from the pNP substrate [32,33,34]. β-d-glucosidase was evaluated with semi-purified (crude) enzyme extracts from either cell lysis or the microbial media. β-d-glucosidase from the five *Aspergillus* strains was identified in the cell culture supernatant. However, the β-d-glucosidase activities of the six probiotics were detected in the cell-free extract (intracellular enzyme) produced by cell disruption. β-d-glucosidase activities were not detected without cell breakage processes or from cell culture supernatants. These results are similar to our previous reports [33,35]. Chi et al. [35] and You et al. [33] found β-d-glucosidase activities in cell-free extracts of *Bifidobacterium bifidum*, *Lactobacillus delbrueckii*, and *Lactobacillus cacei*. A sonication cell disruption process was critical to exhibit β-d-glucosidase’s activities.

**Figure 1:**
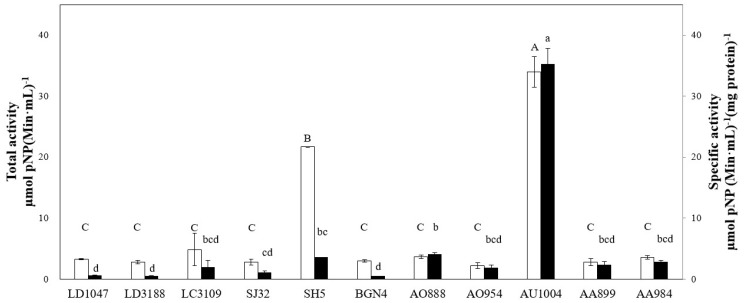
Total (□) and specific (■) β-d-glucosidase activities of 11 cell strains. One-way ANOVA followed by a Tukey’s post hoc test statistical analyses were performed. Treatments with different letters (where A > B > C and a > b > c > d) are significantly different at *p* < 0.05.

The cultures used in this study showed different total and specific β-d-glucosidase activities, which ranged from 0.3 to 145.1 µM pNP (min·mL)^−1^ and 0.5 to 150.0 µM pNP (min·mL)^−1^·(mg of protein)^−1^. Nine microbial extracts—*Lactobacillus delbrueckii* ssp. *delbrueckii* KCTC 1047 (LD1047), *Lactobacillus delbrueckii* subsp. *bulgaricus* KCTC 3188 (LD3188), *Lactobacillus cacei* KCTC 3109 (LC3109), *Bifidobacterium* sp. SJ32 (SJ32), *Bifidobacterium bifidum* BGN4 (BGN4), *Aspergillus oryzae* KFRI 888 (AO888), *Aspergillus oryzae* KFRI 954 (AO954), *Aspergillus awamori* KFRI 899 (AA899) and *Aspergillus awamori* KFRI 984 (AA984)—failed to perform the desired reaction. Two isolates of *Bifidobacterium* sp. SH5 (SH5) and *Aspergillus usamii* KFRI 1004 (AU1004) successfully performed the desired reaction. Figure 1 shows the β-d-glucosidase activities of the tested groups. 

The enzyme activities extracted from the nine strains (i.e., LD1047, LD3188, LC3109, SJ32, BGN4, AO888, AO954, AA899 and AA984) mentioned above were not statistically different (*n* = 3 *p* > 0.05). The β-d-glucosidase activities of AU1004 and SH5 were significantly higher than the other groups (*n* = 3, *p* < 0.001). AU1004 had the highest total and specific activity values (*n* = 3, *p* < 0.001) compared to the others. Accordingly, the fungus AU1004 was utilized in subsequent experiments.

### 2.2. Enzyme Induction

**Figure 2:**
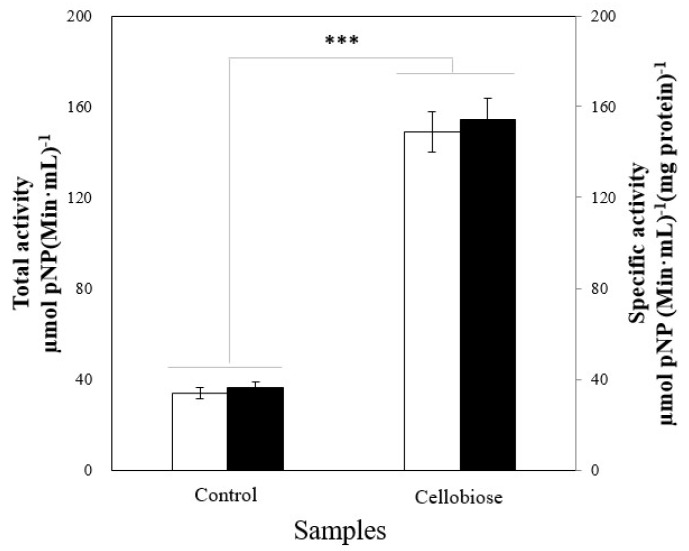
The significantly enhanced total (□) and specific (■) β-d-glucosidase activities via cellobiose treatment of the culture media (*n* = 3). Bars with *** represent statistically significant differences between groups at *p* < 0.001.

We further examined the effect of cellobiose as an enzyme inducer via enzyme activity analysis when AU1004 was cultured in basal medium (BM, cellobiose-free condition) and the experimental condition. As indicated in Figure 2, using cellobiose as the major carbon source in AU1004 cultivation significantly enhanced the β-d-glucosidase activities of AU1004 (*n* = 3, *p* < 0.001). 

β-glucosidase breaks down the β-glucosidic bonds between aglycons and glucose molecules. This enzyme also catalyzes cellobiose into two glucose molecules via β-glucosidic bond hydrolysis [36]. β-d-glucosidase is produced by multiple bacterial and fungal species [37]. The induction of increased β-d-glucosidase may occur when probiotic bacterial and fungal strains are grown in a culture media supplemented with cellobiose. Microorganisms may produce β-d-glucosidase to convert the cellobiose contained in the medium into an energy source. Multiple groups have reported that carboxymethyl cellulose, cellobiose, and Solka-Floc were effective inductors of enzyme biosynthesis [22,38,39,40]. Our previous work showed that *Lactobacillus delbrueckii* Rh2 (cultivated in media containing galactose without glucose) significantly increased β-glucuronidase activity [20]. *Lactobacillus rhamnosus* GG cultivated in media containing 2% cellobiose (*w/v*) as a key carbon source showed significantly enhanced β-d-glucosidase activity compared to media containing glucose [22].

Microorganisms’ sugar ingestion methods vary based on the proteins and enzymes involved in sugar hydrolysis [41]. Such diverse mechanisms support the cell’s effective uptake of multiple sugars to maintain their metabolism, in particular for microbial proliferation under low-glucose environmental conditions [42]. The microorganism’s catabolite repression system of sugar substances is a physiologically-controlled mechanism for the efficient exploitation of carbon sources for microbial growth. This repression system acts as an autoregulatory tool that screens favored substrates (i.e., monosaccharides) within extracellular fluids. Specifically, the utility of various sugars in culture media is regulated by carbohydrate catabolic genes and/or operons at the level of carbon source-specific induction [33,43,44]. In this system, sugars act as inducers and/or repressor of gene expression. The induction-repression mechanism turns on the sugar-specific catabolic genes required to grow in particular conditions. 

Previous studies have used nutrient-dependent enzyme induction systems that provide various carbohydrates in glucose-free culture media. According to Saloheimo et al. [45], glucose media supplements reduced cellulase gene (i.e., *cbhl*, *cbh2*, *egll*, *eg12* and *egl5*) expression of *Trichoderma reesei*. Suto and Tomita [46] summarized the glycosylase induction and catabolic repression models of *Hypocrea jecorina* and *Penicillium purpurogenum*. These two fungal species regulated and produced glycosylases at the transcriptional level to feed carbon sources, multiply in nature, and produce glycosylases. The production steps are as follows: (i) expression at a basal level that mainly produce cellulose to degrade complex plant cellulose near fungal hyphae into cellooligosaccharides; (ii) active cellulase gene transcription by sophorose and gentiobiose that act as cellulase inducers; (iii) full-scale cellulase secretion outside of hyphae via sophorose and glucose production; (iv) active cellulose degradation results in increased cellooligosaccharides and glucose levels; and (v) catabolite repression caused by glucose and decreased cellulase production. According to Ku et al. [21], α-l-arabinofuranosidase and α-l-arabinopyranosidase activity increased simultaneously when *Bifidobacterium longum* RD47 was cultured in a medium supplemented with 2% ascorbic acid (*w*/*v*). The enhanced enzymes were used to catalyze ginsenosides. *Bifidobacterium longum* RD47 also showed increased α- and β-galactosidase productivity via soybean oligosaccharide treatment [47]. 

### 2.3. Biotransformation of Platycoside E and Platycodin D3 into Platycodin D

**Figure 3:**
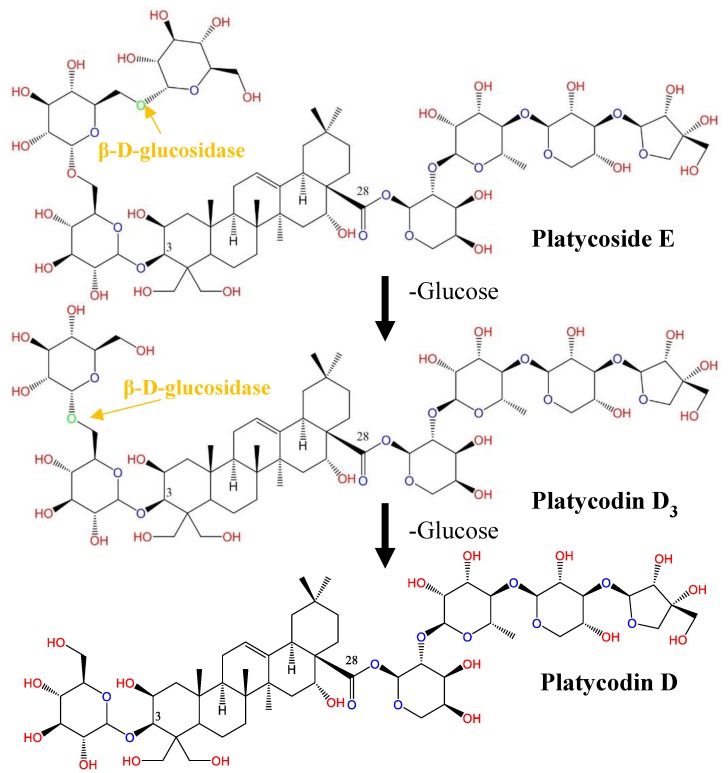
Biosynthetic platycodin D (PD) pathway from platycoside E (PE) and platycodin D3 (PD3) in Platycodi radix (PR). PD and PD3 undergo glycosylation by β-d-glucosidase and transform into PE.

PR extracts contain about 2% platycosides [5]. Among the various platycosides, the ratios of PE, PD3, and PD are approximately 8.3, 15.4 and 20.9%, respectively [48]. These three platycoside types are distinguished by their glucose units present at the C-3 position of PD [5]. Therefore, the transformation of the two major platycosides (i.e., PE and PD3), which account for more than 20% of all platycosides, into a more bioactive platycoside such as PD has significant nutraceutical and pharmaceutical applications. It has been reported that β-d-glucosidase converted PE and PD3, which removed one and two glucose molecules at C-3 of PD [5]. Specifically, β-d-glucosidase hydrolyzed PE along the following pathway: PE→PD3→PD. PD3 was also transformed to PD via the removal of one glucose molecule (Figure 3).

However, a few reports incorporated enzymatic biocatalysis in the preparation of PD [5,26,27]. Prior to our work, various commercially-available enzymes extracted from almonds, microorganisms, and snail digestive tracts were utilized to increase the PD level in PR extracts. However, these biocatalytic processes were time-consuming (1–3 days) or yielded low conversion rates. Specifically, Ha et al. [5] attempted to transform PE and PD3 in PR extracts using two types of commercially-available, almond-derived β-d-glucosidase and one fungal cellulase to increase platycodin D production. Despite β-d-glucosidase treatment for 24 h, most of the platycoside E and platycodin D3 glycosidic linkages at C-3 residues remained intact. The platycodin D content increased by less than 10% after 24 h. They also utilized commercially available fungal cellulase to hydrolyze platycoside E and platycodin D3. Cellulase from *Trichoderma reesei* successfully cut the β-1-6 glucose bonds of platycoside E and platycodin D3 and converted all platycoside E and platycodin D3 (100%) in Platycodi radix extracts into platycodin D after 24 h incubation. Li et al. [26] also reported the transformation of platycoside E and platycodin D3 to platycodin D by commercially available snailase (i.e., a mixture of cellulase, hemicellulase, pectinase and β-glucuronidase) treatment. While their snailase presented notable transformation rates (about 100%), a lengthy incubation process (24 h) was necessary to achieve 100% platycoside E and platycodin D3 transformation. Recently, Lee et al. [27] collected crude enzymes from *Cyberlindnera fabianii* and *Trichosporon loubieri* after cell screening about 200 Korean nuruk microorganisms and attempted to produce PD from Platycodi radix extracts. Their three-day enzyme reaction increased the total amount of PD by between 12% and 43% and featured low selectivity.

**Figure 4:**
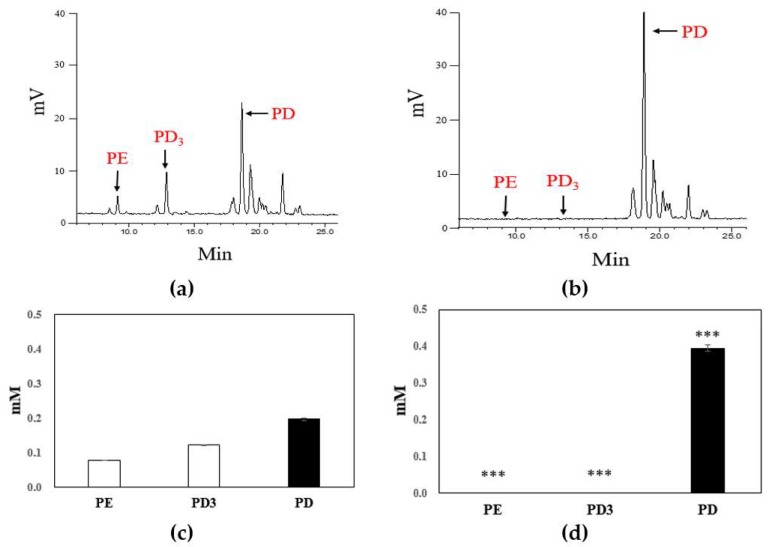
Typical High Performance Liquid Chromatography (HPLC) metabolic profiles of 3 mg crude platycosides separated from PR extracts (**a**,**c**) after a 2 h incubation with AU1004 crude β-d-glucosidase (**b**,**d**). A significantly altered biotransformation profile, with increased PD (■) and reduced PE and PD3 (□), was observed following the enzyme treatment (**c**,**d**). Bars with *** represent statistically significant differences between groups at *p* < 0.001 (*n* = 3, **c** and **d**).

To determine whether our screened extracellular enzyme stimulates the cleaving of platycoside glycosidic bonds, we applied the AU1004 enzyme to PR extracts containing natural substrates (i.e., platycoside E and platycodin D3). The enzymatic reactions of each key point were examined via HPLC analysis; chromatographic data are shown in Figure 4a,b. Within 2 h, >99.9% of PE and PD3 was converted to PD. PE and PD3, which were present at concentrations of 0.08 and 0.12 mM, respectively (*n* = 3, *p* < 0.001) (Figure 4c), were successfully converted to PD within 2 h by AU1004 enzyme incubation. As a result, the PD present at 0.20 mM concentration in the original PR extract increased to 0.40 mM (*n* = 3, *p* < 0.001) (Figure 4d).

**Figure 5:**
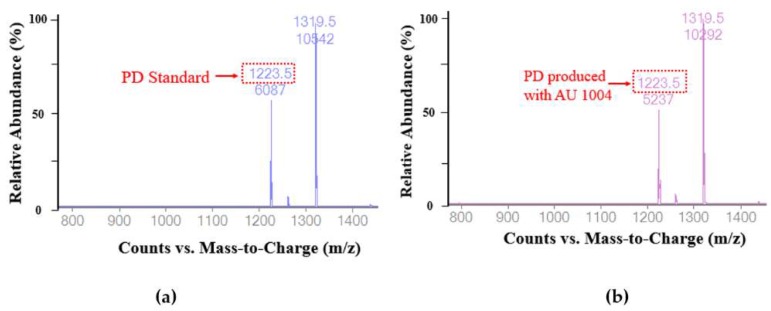
LC/MS profiles in the ESI negative mode of commercially-available, standard PD (**a**) and PD produced by our protocol (**b**). The *m*/*z* ratios of biotransformed PE and PD3 (PD) were 1223.5 and 1319.5 (*m*/*z*), respectively, the same as that of PD. The value of 1319.5 is the resulting adduct sulfate ion [M–H+SO_4_]^−^.

The PD generated via bioconversion was further verified using LC/MS. Specifically, the chemical structure of PD existing in compound peak fractions that were collected and recovered by prep-HPLC was analyzed by Eletrospray ionization (ESI)-Triple Quadrupole LC/MS according to the manufacturer’s instructions. Initially, the ESI-MS experiments were performed in the negative mode to determine molecular weight. The ESI mass spectra of transformed PE, PD3, and standard PD in negative ion mode displayed sharp and distinguished peaks at *m*/*z* 1223.5 and 1319.5, which represent the deprotonated ion [M − H]^−^ of PD (Figure 5a,b). The value 1319.5 is the adduct sulfate ion [M–H+SO_4_]^−^. These results confirm that platycodin D is rapidly generated by the enzymatic hydrolysis of the 1-6 glycosidic linkage at the C-6 of glucose within 2 h. 

### 2.4. The Inhibitory Effects of Platycodin D on LPS-Induced IL-6 and Nitrite Production in RAW 264.7 (KCLB 40071) Mouse Macrophages

To evaluate LPS-induced macrophage inflammatory responses, we investigated the inhibitory effects of platycodin D on LPS-induced IL-6 and nitrite production in RAW 264.7 (KCLB 40071) mouse macrophage cells. Nitrite and IL-6 are commonly used endogenous markers for the characterization of inflammatory diseases, so the measurement of nitrite and IL-6 metabolites in cell lines is considered to be a useful in vitro inflammation monitoring measure [49]. The RAW 264.7 cell lines were incubated with multiple levels of PD produced by our protocol in the presence and/or absence of *Escherichia coli* O127:B8 LPS. The released extracellular nitrite and IL-6 were examined. 

**Figure 6:**
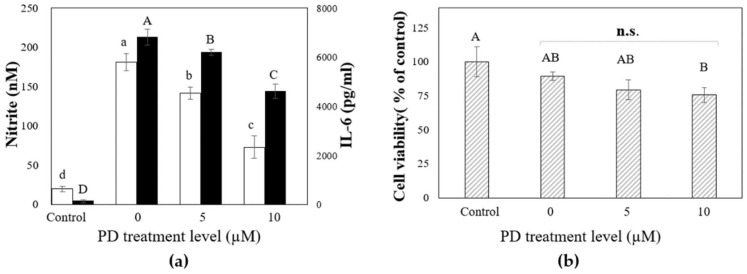
PD treatment regulation of IL-6 and nitrite in macrophage cells. (**a**) The results indicate that PD inhibited the production of LPS-induced IL-6 (■) and nitrite (□) in a dose-dependent manner. The significantly decreased extracellular IL-6 and nitrite levels confirmed the anti-inflammatory effect of PD. The values presented are the mean ± SD of three independent experiments. Bars labeled by letters (where A > B > C >D and a > b > c > d) are statistically significant from each other (*p* < 0.05), which was determined using a one-way ANOVA followed by Tukey’s post hoc test; (**b**) Cell viability (▨) was determined via MTT assay. “n.s.” indicates no statistically significant difference among the three treatment groups with *p* > 0.05 (*n* = 3). Bars labeled by letters (A, AB, B)

Figure 6a shows the effect of PD on the LPS-induced macrophage inflammatory response. The mouse macrophages were treated with the indicated levels of PD for 2 h, and then treated with LPS 0.1 µg/mL for 18 h. The PD-free group was used as an inflammatory positive control. LPS significantly activated RAW 264.7, which secretes inflammatory substances (i.e., IL-6 and nitrite), as shown in Figure 6a (*n* = 3, *p* < 0.001). However, the PD pretreatment prominently downregulated LPS-induced IL-6 and nitrite release in a dose-dependent manner. LPS and PD were not added to the control group. Significantly reduced concentrations of IL-6 and nitrite generated from macrophages by LPS are presented as histograms in Figure 6a (*n* = 3, *p* < 0,001). 

3-(4,5-Dimethylthiazol-2-yl)-2,5-diphenyltetrazolium bromide (MTT) assay results showed no obvious cytotoxicity of PD (up to 10 μM) toward mouse macrophage cells in the presence of LPS although a trend toward decreased cell viability was observed (Figure 6b (*n* = 3, *p* > 0.05)). The findings of this work concur with the previous data and suggest that PD inhibited inflammatory marker production in RAW 264.7 cells [49,50,51,52,53]. 

## 3. Materials and Methods 

### 3.1. Chemicals and Reagents

PE, PD3, and PD were generously donated by BNC Biofarm (Seoul, Korea). The analytical reagents and solvents used in this work were obtained from Samchun Chemical Co., Ltd. (Seoul., Korea) unless mentioned otherwise. All macrophage cultivation materials, including the serum-free cell culture media, fetal bovine serum, and related culture solutions, were GIBCO products from Thermo Fisher Scientific (Waltham, MA, USA). HPLC-grade mobile phases (e.g., acetonitrile, methanol, and water) were purchased from Fisher Scientific (Pittsburgh, PA, USA).

### 3.2. Preparation of Bacterial Crude Enzymes

Lactobacillus delbrueckii ssp. delbrueckii KCTC 1047 (LD1047), Lactobacillus delbrueckii subsp. bulgaricus KCTC3188 (LD3188), Lactobacillus cacei KCTC 3109 (LC3109), Bifidobacterium sp. SJ32 (SJ32), Bifidobacterium sp. SH5 (SH5), and Bifidobacterium bifidum BGN4 (BGN4) were generously donated by BIFIDO LTD (Hongcheon, Korea). The six probiotic strains were incubated in Man–Rosa–Sharpe (MRS) medium (BD Difco™, Franklin Lakes, NJ, USA) broth containing 0.05% (w/v) l-cysteine under strict anaerobic culture conditions for 24 h at 37 °C and subcultured in 50 mL of MRS broth. Cultured probiotic cells were recovered via centrifugation (3000× g for 30 min at 4 °C). The harvested cells (8 log colony-forming unit (CFU)/mL) were washed twice with 50 mM phosphate buffer (PB, pH 6.0, 0.02 M) to remove the media ingredients from the cells. The washed cell samples were then resuspended in 10 mL of phosphate buffer. β-d-glucosidase from the above six probiotic strains was considered as a cytosolic (intracellular) enzyme due to the absence of enzyme activities in the cell-free media supernatant. All probiotic strains were disrupted via cell sonication (Sonics and Materials, Inc., Newtown, CT, USA) at 45 amplitude for 30 min at 4 °C (1 s burst and 1 s cooling intervals) to extract intracellular enzymes for the purpose of producing a crude enzyme solution as described in You et al. [54].

### 3.3. Preparation of Fungal Crude Enzymes

All of the experimental *Aspergillus* strains (*Aspergillus oryzae* KFRI 888, *Aspergillus oryzae* KFRI 954, *Aspergillus usamii* KFRI 1004, *Aspergillus awamori* KFRI 899 and *Aspergillus awamori* KFRI 984) were purchased from the Korea Food Research Institute (Seongnam, Korea). *Aspergillus* crude enzymes were prepared following the methods of You et al. [33] Specifically, the spores were harvested from a subcultured, commercially available potato dextrose agar (glucose 20 g/L, agar 15 g/L and potato extract 4 g/L; Sigma-Aldrich, St. Louis, MO, USA) plate by scraping, followed by suspension of the fungal spores (0.9% NaCl solution with 0.005% polysorbate 80). A hemocytometer (Paul Marienfeld GmbH & Co. KG, Lauda-Königshofen, Germany) was used to count the *Aspergillus* spores. The recovered fungal spores were spiked at 6 log spores/mL (9 log cell spores/L) into the BM with cellobiose (5 g/L). The BM composition was identical to that used in our previous study (You et al.): sodium nitrate (CAT 7631-99-4, 0.5 g/L), dipotassium phosphate (CAT 7758-11-4, 1.0 g/L), magnesium sulfate heptahydrate (CAT 10034-99-8, 0.5 g/L), iron(II) sulfate heptahydrate (CAT 7782-63-0, 0.01 g/L) and 0.5% (*w*/*v*) casamino acid (65072-00-6), with a pH adjusted to 7.0. 

According to Decker et al. [55], *Aspergillus* strains produce extracellular enzymes, so we attempted the concentration and recovery of extracellular β-d-glucosidase from the culture media. All *Aspergillus* strains were incubated in 1 L culture broth at 30 °C for seven days under aerobic conditions with shaking at 180 rpm (SWB-03, JEIO TECH, Anyang, Korea). The mycelia were removed from the culture broth via microfiltration using a 1.6 μm cut-off GF/A borosilicate microfiltration membrane (Z242128, Whatman^®^, Kent, UK). The filtrate-reserving extracellular enzymes were concentrated via a centrifugal membrane module (Amicon Ultra, 30 K molecular weight cut-off, UFC903008, Millipore Korea, Seoul, Korea) at 4000× *g* for 30 min at 4 °C. The concentrated and recovered filtrates were washed and rinsed with the same volume of PB (0.02 M, pH 6.0) to purify the enzymes and remove the media ingredients with a pH adjustment. The washed sample was concentrated a second time using centrifugation with Amicon Ultra centrifugal filter units. The concentrated crude enzyme sample was resuspended in 50 mL of PB.

### 3.4. Assay of β-d-Glucosidase Activity

The β-d-glucosidase activity was determined in the supernatant of the five *Aspergillus* culture media for the fungal extracellular enzyme and in the disrupted cell suspensions of six probiotic strains for the intracellular enzyme. The total bacterial and fungal β-d-glucosidase activities were determined as described by Wie et al. [34]. Specifically, to enumerate the microbial β-d-glucosidase activities, 20 µL of crude enzyme extracts were added to 20 µL of the artificial substrate (5 mM, pNP, # N7006, Sigma-Aldrich, St. Louis, MO, USA) with 60 µL of PB (0.02 M, pH 6.0). The crude enzyme and pNP substrate mixtures were incubated at 37 °C for 8 min with shaking. The enzyme hydrolysis reaction was stopped by adding 100 µL Na_2_CO_3_ (0.5 M). The released p-nitrophenol (pNP) was measured at 450 nm (96 cell microplate reader, Bio-Rad Laboratories, Philadelphia, PA, USA). Specific activity was determined per mg protein. The specific activity (U/mg) of β-d-glucosidase was calculated as units of enzyme activity per mg of protein. The level of protein was evaluated using a commercially available BCA Protein Assay Kit (Pierce™, CAT 23225, Waltham, MA, USA).

### 3.5. Extraction of Crude Platycosides from Platycodi Radix

*Platycodon grandiflorum* root (Platycodi radix, 3 years) powder was purchased from a local grocery market (Seoul, Korea). To extract the platycosides, we used the protocol of Ha et al. [5]. Specifically, 100 g of Platycodi radix powder was added to 1 L of 70% methanol at held at 70 °C in a waterbath shaking at 100 rpm for 4 h. The powder was then removed by filtration (Whatman^®^ Grade 3, Z240478, Maidston, UK). After a dead-end filtration process, the collected filterates were evaporated using an Eyela rotary evaporator N-100 (Rikakikai Co., Ltd., Tokyo, Japan) and 100 mL of deionized water was added to the samples. 

According to Nyakudya et al., Platycodi radix is composed of approximately 90% sugar (i.e., glucose, fructose, saccharose, kestose, inulin and platycodin), 2.4% protein, 2% platycosides, 0.1% lipid and 1.5% ash [1]. It was therefore assumed that the collected sample contains not only platycosides but also other molecules (e.g., soluble sugars, dietary fibers and proteins). A purification process was conducted via absorption chromatography to remove the unwanted compounds in the sample. Specifically, the sample was loaded onto a column (100 cm × 5 cm) packed with non-polar copolymer styrene-divinylbenzene adsorbent resin (Diaion^®^ HP-20, 9052-95-3, Sigma-Aldrich, St. Louis, MO, USA) and sequentially eluted with water, 30, 70, and 100% methanol so that the platycosides and other molecules were separated by polarity differences. According to Ha et al. [5], most platycosides exist in the 100% methanol fractions, so we only used the sample collected from the 100% methanol fraction for this study. Using a rotary evaporator, the 100% methanol fraction was evaporated and the sample residues (powder form) were used as crude platycoside samples [5].

### 3.6. Biotransformation of Platycosides Using β-d-Glucosidase of A. usamii

The biotransformation of platycosides was conducted based on the procedures of You et al. [33]. Three milligrams of crude platycosides were resuspended in 1 mL of *A. usamii* crude enzyme extract and incubated at optimal pH and temperature conditions (6.0 and 40.0 °C) in a waterbath shaking at 180 rpm for 2 h. The optimal pH and temperature of crude β-d-glucosidase of *A. usamii* by pNP-assay was used for the rapid bioconversion process. The *Aspergillus* enzyme activities in each experiment were evaluated by measuring the product level (i.e., free *p*-nitrophenol molecules). pNP was used as a substrate, and the level of released free *p*-nitrophenol molecules was estimated by reading the absorbance at 405 nm using a microplate reader (Bio-Rad Laboratories, Philadelphia, PA, USA). The biotransformation process was terminated by boiling (100 °C) for 10 min. The collected samples were freeze-dried and stored at −20 °C before use. The residue was dissolved in 3 mL of methanol for high-performance liquid chromatography (HPLC) with an evaporative light scattering detector (ELSD) [33,46].

### 3.7. Analysis of PE, PD3 and PD via HPLC-ELSD

The platycoside-methanol solution was centrifuged for phase separation. The supernatant was collected and microfiltered by a Millex LCR syringe filter membrane (0.45 μm cut off, Millipore, Bedford, MA, USA). Twenty microliters of the filtered sample were loaded on a HPLC system. HPLC analysis was conducted with a Dionex P680 HPLC (Dionex Corporation, Sunnyvale, CA, USA) equipped with a ZORBAX^®^ SB C18 reverse phase 5 μm, 4.6 mm × 250 mm column (Agilent Technologies, Santa Clara, CA, USA) and PL-ELS 2100 ELSD (Dionex Corporation, Sunnyvale, CA, USA). A solvent gradient consisting of water with a pH of 2.5 (0.1% trifluoroacetic acid) (A) and acetonitrile (B) flowing at 1.0 mL/min under the following profile was used: 0–10 min (25–30% B), 10–20 min (30–45% B), 20–40 min (45–100% B), and then equilibrated with 100% B for 10 min. The ELSD was set to a probe temperature of 70 °C, a gain of 7, and the nebulizer gas nitrogen adjusted to 2.5 bar. The chromatogram peaks were assigned by comparing to the retention times of PE, PD3, and PD standard compounds under the same conditions.

### 3.8. Preparation of Platycodin D Using Preparative-HPLC

To separate and recover the purified platycodin D molecules from the platycoside-methanol solution, preparative HPLC (Young Lin Acme 9000, Younglin Instrument Co., Ltd., Anyang, Korea) equipped with a semi-preparative ZORBOX SB-C18 5 μm, 9.4 mm × 250 mm column (Agilent Technologies, Santa Clara, CA, USA) was utilized. The mobile phase consisted of solvent A (0.1% (*v*/*v*) trifluoroacetic acid in HPLC grade water, pH 2.5) and solvent B (methanol) with the following gradient: 0–20 min, linear gradient from 55% to 65% B; 20–35 min, 65–70% B, 35–50 min, 70–100% B. The injection volume of the samples was 1 mL, the flow rate was 5 mL/min, and the absorbance was measured using a UV detector (UV VIS detector, Younglin Instrument Co., Ltd., Anyang, Korea) at a wavelength of 210 nm.

### 3.9. Chromatographic and Mass Spectrometric Conditions

The sample molecular weights were determined using an Agilent 6410A triple quadrupole LC/MS system (Agilent Technologies, MA, USA) equipped with a ZORBAX^®^ SB C18 column 5 μm, 4.6 mm × 250 mm column (Agilent Technologies, Santa Clara, CA, USA) to qualitatively confirm that the platycodin D molecules were purified in the samples collected after the preparative-HPLC process. Mass spectrometric analysis of the platycodin D in the sample was carried out at the Central Laboratory for Instrumental Analysis at Kyung Hee University Global Campus (Youngin, Korea).

### 3.10. Evaluation of Anti-Inflammatory Effects of Platycodin D

We conducted an in vitro assay using macrophages to proceed with the qualitative evaluation and confirmation of functionality of our platycodin D. Commercially available mouse macrophage cell lines (RAW 264.7, KCLB 40071) were used to evaluate the anti-inflammatory effects of platycodin D via nitrite and IL-6 assays. Mouse Macrophage Cell Lines (RAW 264.7, KCLB 40071) were purchased from the Korean Cell Line Bank (Seoul, Korea). The cell lines were cultured under identical culture conditions, and the cell bioavailability was maintained by MTT assay according to the manufacturer’s instructions. The cell lines were subcultured in Dulbecco’s modified Eagle’s medium (GIBCO, 12491-023, Carlsbad, CA, USA) with 10% (*v*/*v*) fetal bovine serum (GIBCO, 12483-020, Carlsbad, CA, USA) and 1% (*v*/*v*) antibiotic-antimycotic solution (GIBCO, R25005, Carlsbad, CA, USA) at 37 °C in a humidified atmosphere of 95% air and 5% CO_2_. Cell enumeration was conducted using a hemocytometer (Paul Marienfeld GmbH & Co. KG, Lauda-Königshofen, Germany); the number of viable cells was determined using trypan blue dye (Sigma-Aldrich, T8154, St. Louis, MO, USA) exclusion. To evaluate the anti-inflammatory effects of platycodin D, we intentionally treated commercially available lipopolysaccharide (LPS) extracted from *Escherichia coli* O127:B8 (Sigma-Aldrich, L4516, St. Louis, MO, USA) into cell lines with platycodin D molecules and evaluated the secretion levels of nitrite and IL-6. These evaluation protocols were adopted from Kim et al. [56]. Specifically, the macrophage cells were seeded in polystyrene tissue culture-treated, 96-well plates (Corning^®^ 96 Well, #3596, Corning, NY, USA) at 5 × 10^4^ cells/well with 200 µL of complete cell culture media and incubated at 37 °C for 24 h. The cell lines were treated and incubated for 2 h with 5, 10, or 20 µM of platycodin D extracted and purified from our samples. To evaluate the LPS-induced macrophage inflammatory responses, LPS (0.1 µg/mL) was treated and incubated for 18 h. Sample centrifugation (Hanil Scientific Inc., Hanil Union 32R PLUS, Kimpo, Korea) was carried out at 100× *g* for 5 min at 4 °C to obtain the medium supernatant from which the macrophages had been removed. The supernatants were used for the subsequent nitrate and IL-6 assays. The nitrite and IL-6 supernatant concentrations (100 µL/well) in the medium were measured using a commercially available colorimetric nitrite/nitrate assay kit (Sigma-Aldrich, #23479-1KT-F, St. Louis, MO, USA) and IL-6 assay kit (BD OptEIA™ Mouse IL-6 ELISA Kit, 550950, BD Pharmingen, San Diego, CA, USA). The analysis process was conducted according to the manufacturer’s instructions. The nitrite and IL-6 levels were measured at 540 and 450 nm using a microplate reader (Bio-Rad Laboratories, Philadelphia, PA, USA), respectively [47]. Cell viability was determined using the MTT Cell Proliferation Kit I (11465007001 ROCHE, #11465007001, Basel, Switzerland) according to the manufacturer’s instructions. 

## 4. Conclusions

The objective of this work was to use edible bacterial and fungal strains (i.e., *Bifidobacterium*, *Lactobacillus* and *Aspergillus* spp.) to biotransform platycoside-enriched PR extracts and increase the level of PD, which likely confers biofunctionality to consumers. The results of this work indicate that among the 11 microorganism strains tested, AU1004 had the highest β-d-glucosidase activity and active platycoside E and platycodin D3 bioconversion properties, with a transformation rate of >99.9% within 2 h. As the PD precursors were converted, the PD content increased proportionally. Thus, biocatalysis via AU1004 extracellular enzymes could provide a means to overcome the major factors (e.g., low recovery percentage and time requirements) that limit the development of novel, platycodin D-enriched products. Our method may cut production costs by eliminating the heating, and acid hydrolysis of PD production. Moreover, our study contributes to further investigation of the large-scale production of value-added PR products.

## Abbreviations

PR	Platycodi radix
PE	Platycoside E
PD3	Platycodin D3
PD	Platycodin D
Glc	β-d-Glucopyranose
Api	β-d-Apiofuranose
Xyl	β-d-Xylopyranose
Rha	α-l-Rhamnopyranose
Ara	α-l-Arabinopyranose
pNP	para-Nitrophenol
BM	Basal medium
LPS	Lipopolysaccharide
NO	Nitrite
IL	Interleukin
ELISA	Enzyme-linked immunosorbent assay
MTT	3-(4,5-Dimethylthiazol-2-yl)-2,5-diphenyltetrazolium bromide
